# Biophysical characterization of polyphenol aggregates in *Moringa oleifera* leaves water extract: stability and surface exposure effect on antioxidant activity under dilution

**DOI:** 10.1007/s00249-025-01786-4

**Published:** 2025-09-03

**Authors:** Rita Carrotta, Fabio Librizzi, Vincenzo Martorana, Samuele Raccosta, Maria Rosalia Mangione

**Affiliations:** Institute of Biophysics, National Research Council, Palermo, Italy

**Keywords:** Polyphenols, Folin–Ciocâlteu assay, Moringa oleifera, Self-assembly

## Abstract

The aqueous extract of *Moringa oleifera* leaves has been previously characterized for its polyphenolic composition, yet the behavior of its colloidal aggregates under dilution remains largely unexplored. In this study, we investigate the structural and chemical properties of these aggregates at room temperature, focusing on their stability and surface exposure upon dilution. Although the aggregates break up as dilution increases, they never fully dissolve within the conditions explored. Both multi-angle static light scattering and dynamic light scattering highlight aggregates fragmentation and size heterogeneity under dilution. UV–vis absorption spectroscopic data strongly suggest that the aggregates of different sizes present in the extract are homogeneously constituted, as their spectra are similar to those of the main polyphenol components. The Folin–Ciocâlteu assay reveals an increase in gallic acid equivalent values normalized for extract concentration, suggesting that fragmentation prompted by dilution enhances the exposure of reactive sites. A very basic model, considering only one kind of aggregate with uniform density, is employed to support this interpretation. Assuming this model, the Folin–Ciocâlteu assay data allow to grasp the law regulating the change of the aggregate average size under dilution, i.e., a power law. Additionally, in-liquid atomic force microscopy imaging confirms the presence of smaller but still aggregated particles at high dilution, enabling the calculation of a height distribution, that is consistent with the model prediction. These findings provide insights into the dynamic behavior of polyphenol-rich aggregates in aqueous systems and their potential implications for bioavailability and reactivity.

**Supplementary Information:**

The online version contains supplementary material available at 10.1007/s00249-025-01786-4.

## Introduction

Recently, scientific research has increasingly focused on the study of natural compounds as raw materials with potential applications in the fields of pharmaceutical, cosmetic, food industry, and biomaterial science (Obulesu and Rao 2011; Liu et al. 2017; Xu et al. 2018; Ferreira et al. 2021; Xiao et al. 2023; Rybczyńska-Tkaczyk et al. 2023). In this context, polyphenols have received great attention: polyphenols are natural compounds abundant in fruits, vegetables, and cereals, known for their strong antioxidant and anti-inflammatory properties and their chemical structure prone to self-interaction and functionalization (Tsao 2010; Guo et al. 2016; Zhou et al. 2020). The broad functionality of polyphenol compounds is rooted in their chemical structure. Polyphenols are *π*-conjugated compounds containing OH phenolic groups that can be classified, based on their chemical structure, into four main families: flavonoids, phenolic acids, stilbenes, and lignans. Their potent antioxidant and anti-inflammatory actions are key in lowering the risk of chronic conditions such as diabetes, cancer, cardiovascular diseases, and neurodegenerative disorders, so contributing to disease prevention, supporting cognitive function, and enhancing overall well-being (González-Romero et al. 2022; Rudrapal et al. 2022; Bhat et al. 2022; Rana et al. 2022; Sun and Shahrajabian 2023). Moreover, their capacity to hinder the abnormal aggregation of proteins or peptides is believed to be critical in protecting against neurodegenerative diseases like Alzheimer’s, Parkinson’s, and Huntington’s disease (Shariatizi et al. 2015; Stefanescu et al. 2020; Boubakri et al. 2020; Tinku and Choudhary 2023; Carrotta et al. 2025). These findings reinforce the growing interest in natural polyphenols for both disease prevention and therapeutic applications (Yin et al. 2024). Moreover, numerous bioactive compounds can be recovered from food and agricultural industry waste, supporting the principles of the circular economy (Giacomazza et al. 2018; Rodrigues Machado et al. 2023; Yin et al. 2024; Sabbatini et al. 2024).

In a previous work we evaluated the use of aqueous extracts from Moringa oleifera (MO) as strategic phytotherapy for Alzheimer’s Disease (AD) (Carrotta et al. 2025). MO, often called the “miracle tree,” is a highly nutritious plant widely used in traditional and modern medicine. Its many health benefits have been correlated to the antioxidant and anti-inflammatory effects due to the abundance of polyphenolic fraction (Farooq et al. 2012; García-Beltrán et al. 2020; Arora and Arora 2021; Azlan et al. 2023). Our previous investigation revealed that MO aqueous extracts inhibit Aβ aggregation in vitro, protect human neuroblastoma cells from oxidative stress, and reduce the AD typical cell alterations as observed in an AD cell model. Morphological characterization of aqueous extracts showed the presence of aggregates of different dimensions, depending on the extraction procedure and polyphenols concentration. The occurrence of aggregates suggested a self-organization of polyphenol molecules causing a nonlinear dose dependence behavior of the inhibition ability on Aβ aggregation pathway. Therefore, the present work focuses on the structural characterization of the aggregates spontaneously formed during the aqueous extraction from MO leaves. Folin–Ciocâlteu assay, in-liquid atomic force microscopy acquisitions, and static and dynamic light scattering experiments on serial dilutions of MO aqueous extracts show a progressive fragmentation of the aggregates, coupled with an increase on polyphenols antioxidant activity. This behavior agrees with the polyphenols capacity of self-assembly and opens new biotechnological perspectives in the use of spontaneous polyphenol aggregates as reservoir of bioactive compounds. To the best of our knowledge, this is the first time that fragmentation of polyphenol aggregates has been evidenced by a biophysical characterization and the first time that this mechanism is related to the Folin–Ciocâlteu assay, that clearly shows a nonlinear behavior upon dilution of the analyzed sample.

## Material and methods

### Sample preparation

Dry MO leaves, kindly supplied by a local grower, were crushed in a mortar with liquid nitrogen to obtain a fine powder. For the extraction at room temperature 0.3 g of the powder was mixed with 20 ml of water at room temperature. The sample was sonicated for 20 min at 20 °C and then incubated in the dark at 20 °C for 3 days. At the end of the extraction procedure, the sample was filtered with a gauze to eliminate the coarse particles and then sequentially filtered with 0.8 µm and 0.2 µm filters (sartorius CA). Finally, the extract was aliquoted and stored at − 80 °C until use. Size fractionation of the sample was obtained by using 3 KDa centrifuge filters (Amicon Ultra MWCO 3 KDa Millipore).

### Folin–Ciocâlteu assay

Total phenol content of all the extracts was determined using the Folin–Ciocâlteu assay according to Gutfinger (Gutfinger 1981) with slight modifications. Briefly, MO extract was serially diluted in water up to 32-fold. Then, 20 μL of each dilution was mixed with 480 μL of distilled water and 50 μL of Folin–Ciocâlteu’s reagent. After 3 min 100 μL of Na_2_CO_3_ and 350 μL of water were added. The mixture was allowed to stand for 90 min at room temperature in the dark. The absorbance was measured at $\lambda =765\text{ nm}$ against a reagent blank using a Shimadzu UV-2401 PC spectrophotometer. Gallic acid was used as standard for preparing the calibration curve ranging 50–500 mg/l by using the same above described protocol. The total phenolic concentration was expressed as mg/L gallic acid equivalent (GAE). In order to compare the phenol availability in samples at different dilutions, the values normalized for concentration (GAE_norm_) were obtained by taking into account the dilution factor d, GAE_norm_ = GAE*d, and the phenol content was expressed as mg/L gallic acid equivalent per ml of MO extract. All assays were carried out at least in triplicate.

### UV–vis absorption

Spectra in the UV–Vis range were recorded by a Shimadzu UV-2401 PC spectrophotometer. The solution absorption was measured in a quartz cuvette with a 1 cm path at 20 °C. For the sake of comparison spectra have been registered on samples suitably diluted (20 × for the total and filtrate samples, and 52 × for the retentate one) in the wavelength range 200–600 nm and corrected for the background.

### Static and dynamic light scattering

MO samples at different dilutions were characterized by Multi-Angle Static Light Scattering (MALS) and single-angle Dynamic Light Scattering (DLS) at 20 °C in a thermostated cell compartment of a Brookhaven Instruments BI200-SM goniometer. The temperature was controlled within 0.1 °C using a thermostated recirculating bath. The static scattered light intensity and the intensity autocorrelation function ${g}_{2}\left(t\right)$ were measured by using a Brookhaven BI-9000 correlator and a 20mW HeNe laser at *λ* = 632.8 nm. DLS measurements were performed at 90°, corresponding to *q* = 18.7 μm^−1^. The field autocorrelation function, $g_{1} \left( t \right) = \beta \left[ {g_{2} \left( t \right) - 1} \right]^{{{1 \mathord{\left/ {\vphantom {1 2}} \right. \kern-\nulldelimiterspace} 2}}}$, obtained by DLS was analyzed using a smoothing-constrained regularization method, and as a result, the intensity-weighted size distribution was obtained from the analysis (Stepanek 1993). MALS, that is the static scattered intensity as a function of the wave vector *q*, was measured to obtain the form factor of the aggregates at the different dilutions in the range 5 < *q* < 25 μm^−1^. MALS data were corrected for the solvent background and normalized by using the scattering intensity of the toluene as reference (R_tol_ (632.8 nm) = 14 × 10^−6^ cm^−1^).

### In-liquid atomic force microscopy

30 µL of solutions were deposited onto freshly cleaved mica surfaces (Agar Scientific, Assing, Italy) and incubated for 20 min before rinsing with deionized water. About 200 µL of deionized water was added once the sample was set in the apparatus. Quantitative Imaging AFM measurements were performed by a Nanowizard III scanning probe microscope (JPK Instruments AG, Germany) equipped with a 15 μm z-range scanner, and AC40 (Bruker) silicon cantilevers (spring constant 0.1 N/m, typical tip radius 8 nm). The 2 μm x 2 μm images (resolution 256 × 256 pixels) were acquired with a force setpoint of 150 pN and an extension speed of 25 μm/s (*z*-length 50 nm and pixel time 5 ms). The cantilever was thermally calibrated by using the tool in JPK software (Hutter and Bechhoefer 1993). The AFM images have been analyzed with a home-made program to obtain the height distribution. Gray-scale AFM images were first transformed into 1-bit images by setting a hard threshold (40% of the maximum measured height). On the 1-bit images, approximately circular objects were detected by using circular Hough transform (in particular the “imfindcircles” function in a GNU-Octave environment, with a sensitivity value of 0.95). This procedure resulted in the detection of 210 and 74 circular objects for the undiluted sample and for 300-fold diluted sample, respectively. Since the measure of diameter of the objects is strongly affected by the tip size, we classified each object in the images for its maximum height value, with respect to the background value in the same rows of the images. The background was estimated by sorting pixels in the rows by height and taking the average value of the lowest 25% fraction, with minimal differences (about 0.5 nm) observed when varying this fraction from 1 to 25%. This procedure should limit artifacts due to AFM images acquisition and excludes a significant underestimation of particles height due to the presence of underneath layers of aggregates.

## Results

### Folin–Ciocâlteu assay

To determine the total polyphenolic content of MO extracts we used Folin–Ciocâlteu (FC) assay, a reference method for the quantification of total polyphenols in food. The assay relies on an electron transfer reaction where the antioxidant species serves as the electron donor, while the yellow FC reagent as the oxidizing agent (Pérez et al. 2023). The reduction of FC leads to a color change from yellow to blue. More specifically, when electrons are transferred from phenolic compounds to FC reagent in an alkaline solution, blue complexes are formed which can be measured by absorption at 765 nm. The extent of this color change, once the reaction reaches completion, is linearly related to the reducing power of the phenolic compounds. The antioxidant reducing ability for solution extracted from food is expressed in terms of a standard compound, often the compound of choice being gallic acid (GA). Based on the linearity of the method, GA solutions at different concentrations are used to prepare a calibration curve.

**Fig. 1:**
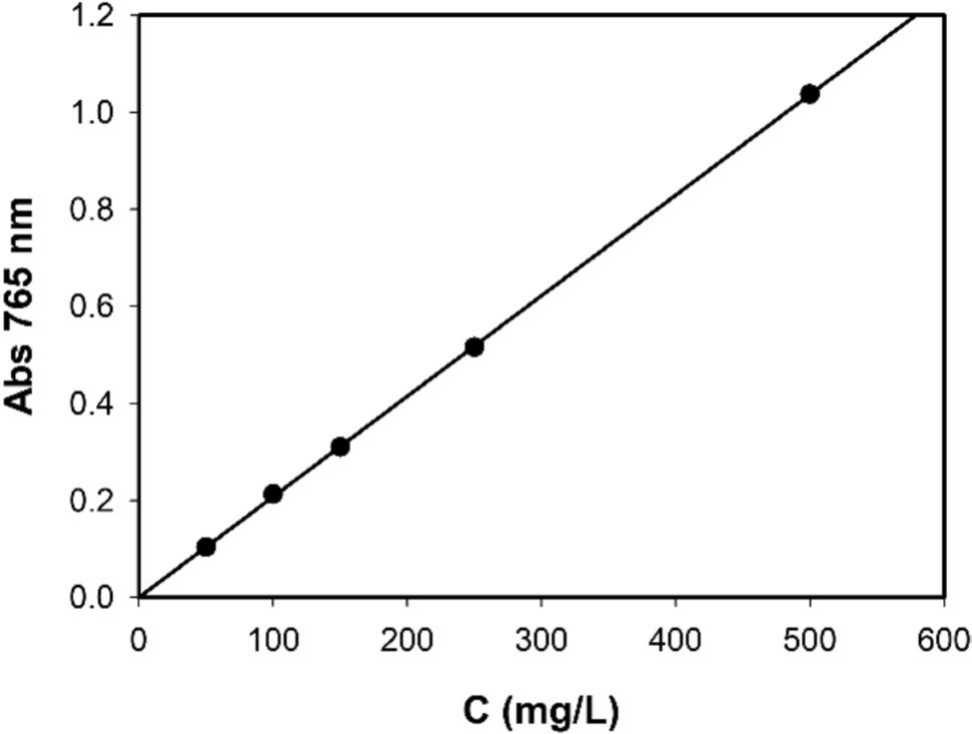
Gallic acid calibration curve. Data errors, coming from triplicate repetition, are smaller than point width

Figure 1 reports the calibration curve obtained from GA solutions at different concentrations ranging from 50 to 500 mg/L, indeed showing a linear behavior. The GAE in MO aqueous extracts can be inferred by using the slope obtained from the linear data fit, as reported in Fig. 1

$$\left( {m = \left( {2.070 \pm { }0.005} \right)*10^{ - 3} { }\frac{{\mathrm{L}}}{{{\mathrm{mg}}}}} \right)$$.

**Fig. 2:**
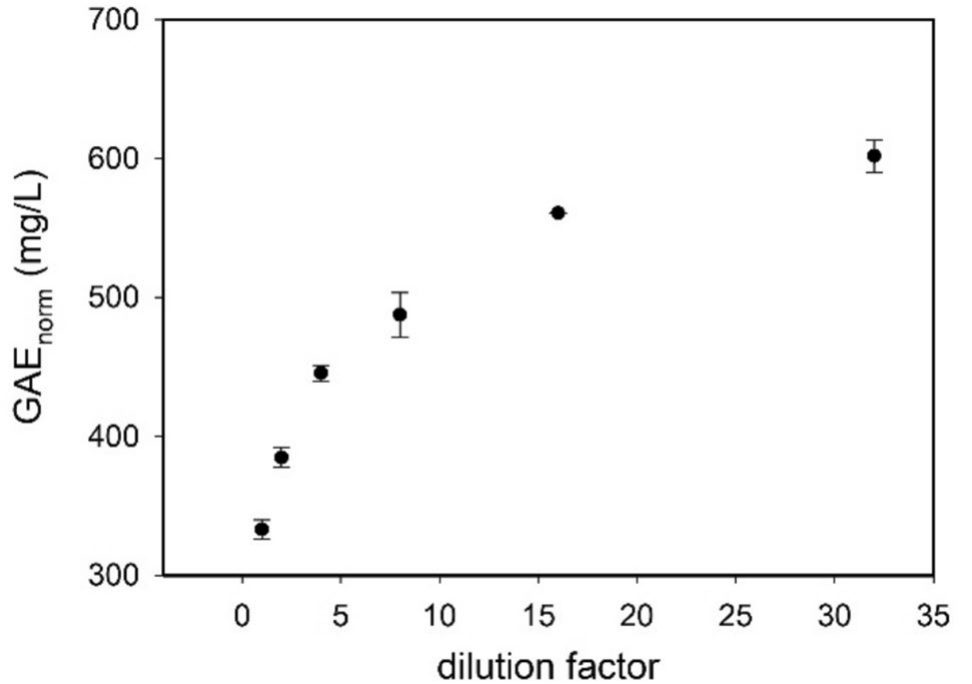
Normalized GAE values for total sample versus different dilution values. Data errors result from a triplicate repetition

In Fig. 2 the total polyphenolic content for MO extracts is reported as the equivalent concentration of gallic acid (by using the method described above). The reported GAE_norm_ values are normalized for the dilution factor and plotted against the dilution factor, in the range 1–32-fold. Unexpectedly, normalized GAE values showed an increase by increasing dilution, thus clearly showing that, as the dilution increases, the relative capacity of the MO extract polyphenol content to fight the FC reagent oxidation grows.

The room-temperature MO extract, as previously investigated by HPLC–MS, presents a high content of polyphenols. Moreover, aggregates of few tens of nanometers were detected by AFM scan on the dried sample, after 60 times dilution (Carrotta et al. 2025). The polyphenols propensity to self-assembly is well documented in literature (Li et al. 2016; Xiang et al. 2017; Zhou et al. 2020; Guo et al. 2021; Han et al. 2022). Based on this, FC results can be interpreted by considering that the increase in antioxidant capacity arises from the disaggregation of unstable aggregates, as dilution increases. Thus, the increase of GAE is correlated with an increased availability of free polyphenols or even with increased solvent exposure of reactive polyphenol groups.

**Fig. 3:**
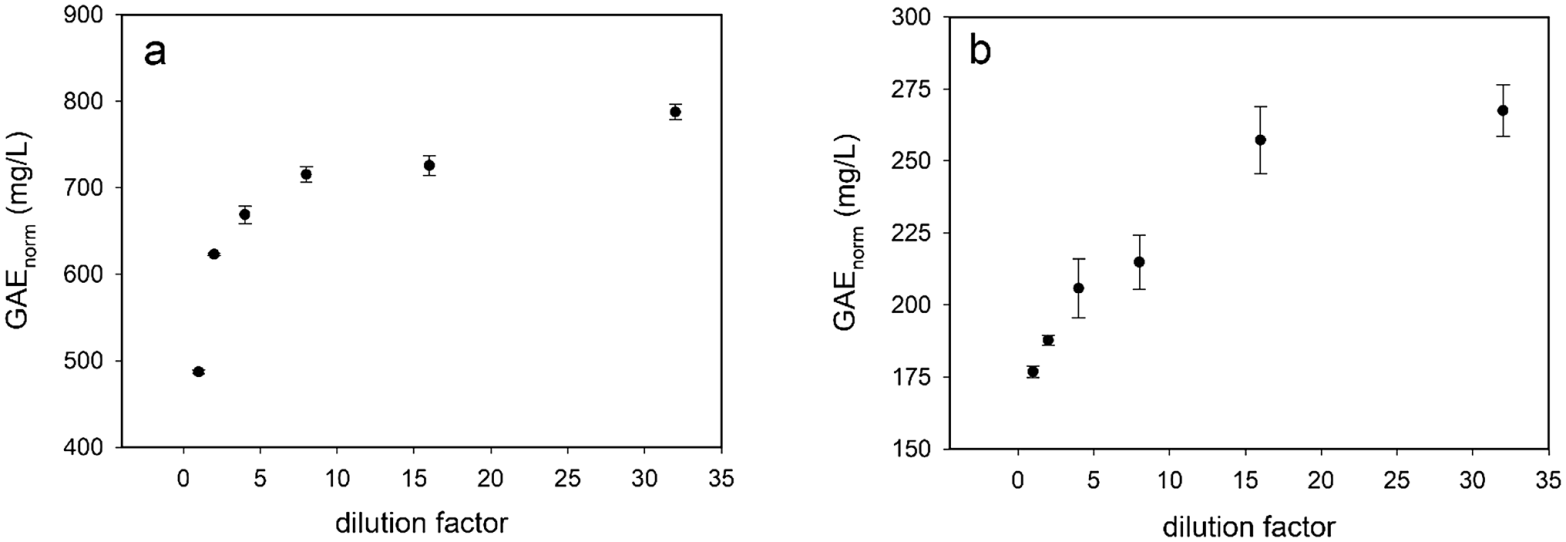
Normalized GAE values versus dilution factor for retentate sample **a** and 3KDa filtered sample **b**. Data errors result from a triplicate repetition

To further explore this scenario, the MO extract was filtered through a 3 KDa cut-off centrifuge filter, in order to separate small species, smaller aggregates and free polyphenols, from the larger ones. Both solutions, the retentate fraction, rich of species with mass larger than 3KDa, and the filtered fraction, were tested for total polyphenol content (TPC), by FC assay in the dilution range 1–32-fold. Results are reported in Fig. 3.

Dilution normalized data show that GAE_norm_ values for both samples, the retentate and the filtrate, depend on the dilution factor, similarly to the total MO extract. Larger values are measured for the retentate sample, in agreement with a higher polyphenolic content present in this sample, rich of species with mass higher than 3 KDa, due to the filtration procedure. The volume of the retentate was in fact 2.6 times smaller than the starting one. Interestingly, also the filtrate sample shows a persistent dependence on dilution, strongly suggesting that even the species with lower mass can further disassemble under dilution.

To obtain a deeper characterization of MO polyphenol species and their tendency to dissociation, different techniques such as UV–Vis absorption, light scattering, and in-liquid AFM were applied.

### UV–vis absorption

**Fig. 4:**
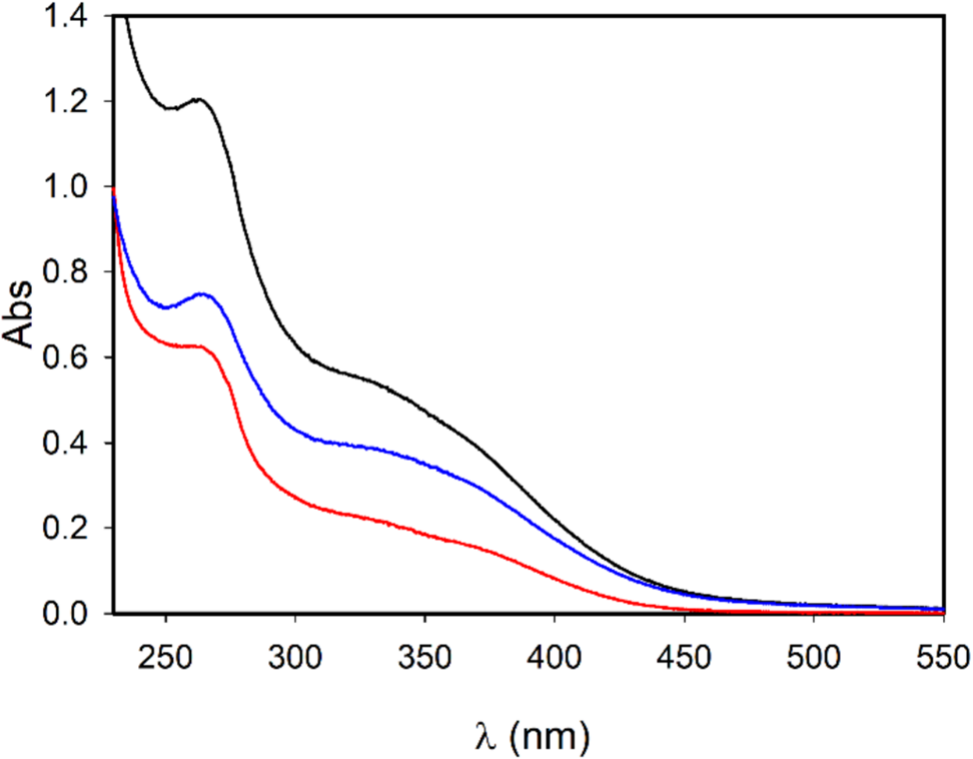
UV–vis spectra for different samples deriving from the MO aqueous extract: total sample (black), retentate sample (blue), 3 KDa filtered sample (red)

UV–Vis spectra for the three samples were acquired to investigate and compare their spectroscopic characteristics. Figure 4 shows a similar shape for spectra from all the samples. The lack of significant differences supports the fact that the main components in all the samples are polyphenols.

Signal spectra are in fact coherent with the main species present, according to HPLC-HESI-MS analysis previously reported (Carrotta et al. 2025). Absorption spectra shown in Fig. 4 are typical of polyphenols, such as quercetin and kaempferol derivatives (Naseem et al. 2004; Anouar et al. 2012; Biler et al. 2017).

### MALS and DLS

**Fig. 5:**
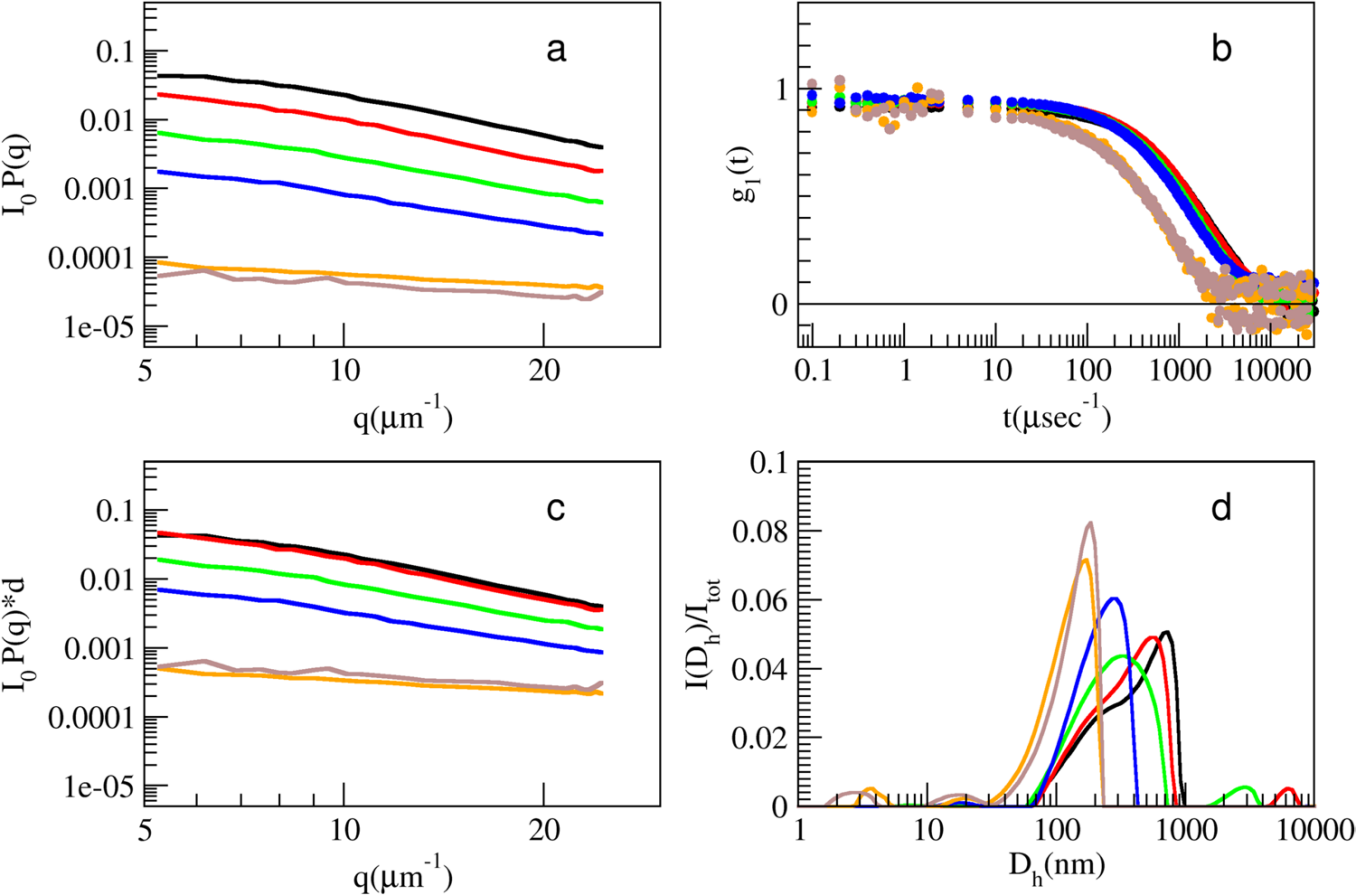
Multi-angle light scattering and dynamic light scattering at 90° for retentate sample at different dilution: starting sample (black), *d* = 2x (red), 3x (green), 4x (blue), 6x (orange), 10x (brown). Absolute (**a**) and concentration-normalized (**c**) scattered intensity. Field autocorrelation function (**b**) and intensity-weighted size distribution (**d**) arising from the analysis of *g*_1_(*t*), according to a CONTIN-like method

Light scattering technique was also used to investigate the effect of dilution on different samples. The scattering from the 3 KDa filtrate sample gave such a low signal to noise ratio that no dilution studies were feasible. The study of the dilution effect was instead possible for the retentate sample. MALS results (Fig. 5a, c) yield a complementary information with respect to the hydrodynamic size distribution obtained by DLS. Static light intensity is practically flat for particles smaller than $\frac{\lambda }{10}$(about 60 nm). For larger particles MALS data are proportional to the form factor $P(q)$, its knee point being related to the intensity averaged particle dimension. When intensity vs. *q* plot displays a curvature, this is in fact a signature of the presence of species larger than 60 nm in the solution (Glatter and Kratky 1982). Dilution of the retentate (> 2 times) brings to a progressive decrease of the normalized intensity. As a matter of fact, if no disaggregation is present, the normalized data (Fig. 5c) should all overlap. Instead, after the first dilution, the larger the dilution is the stronger the decrease and flattening of the *I* vs. *q* data, up to the detection limit reached with the last sample, where the signal is not very different from background. Coherently and independently DLS suggests the presence of small particles, tens of nm wide (Fig. 5 b, d), in agreement with a previous work of the authors, where in-air AFM characterization of the room-temperature extract 60 times diluted was reported (Carrotta et al. 2025). Such particles are probably present also in the more concentrated samples, and a trace appears also in the 4 × dilution, though light scattering is almost blind to those particles, when even small amounts of larger ones (hundreds of nm wide) are present (Berne and Pecora 2000). Taken all together, these results highlight a mechanism in which the particles upon dilution break down into smaller species.

### AFM

In order to complement the evidence coming from FC assay and from light scattering measurements, in-liquid AFM was used to best characterize the samples. Actually, in a previous work in-air AFM characterization of a 60 times diluted room-temperature extract was already reported (Carrotta et al. 2025).

**Fig. 6:**
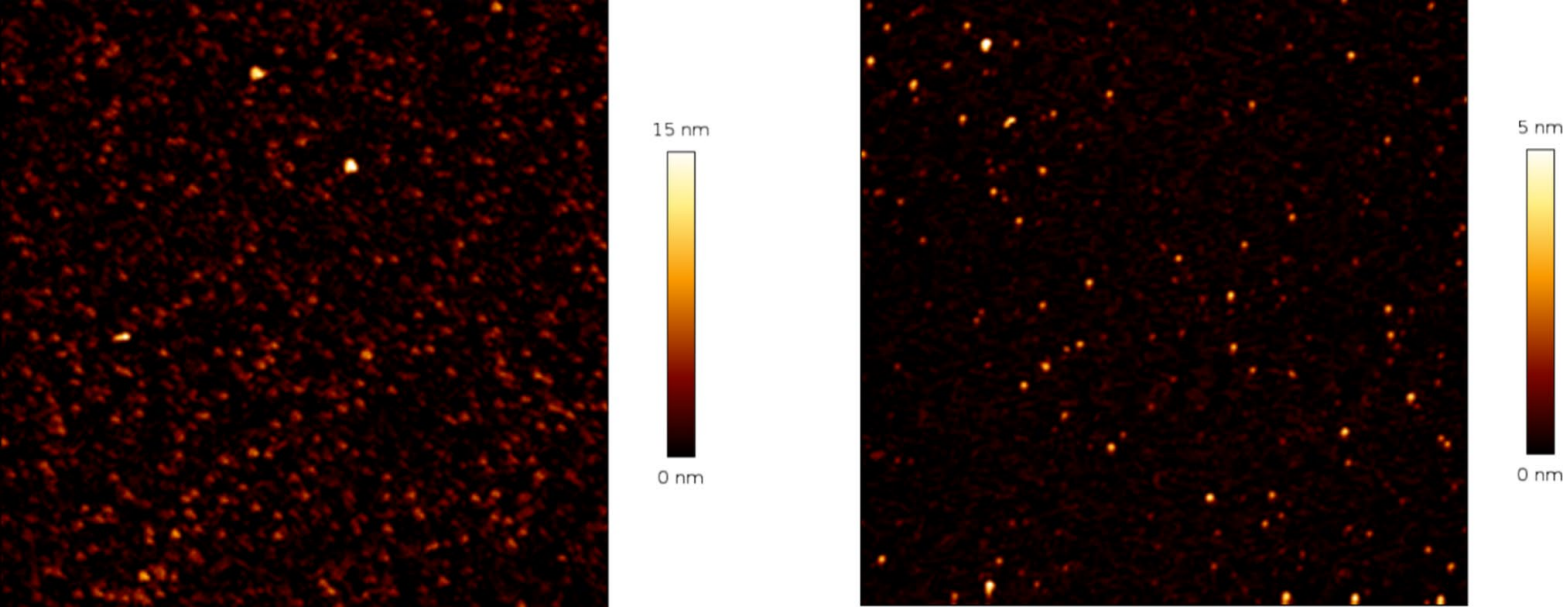
Representative AFM (2 × 2) μm^2^ scans of the undiluted (left) and of the 300 × diluted extract sample (right)

However, since the dry sample treatment could raise some doubts about the actual presence of aggregated species in solution, in-liquid AFM, without and with sample dilution, was performed. The obtained measured height gives an estimate of the size of particles present in the samples and adsorbed on mica. The extract solution is rich in particles having dimensions below about 20 nm (Fig. 6a) and also the presence of few nm large particles is detected, as well as DLS shows. Strong dilution of the sample (300x) leads to a decrease in particle dimensions, below about 8 nm (Fig. 6b). To better evidence this result, Fig.S1 shows the same scan reported in Fig. 6b with the same color height scale as Fig. 6a. This result, again in agreement with DLS size distribution, demonstrates the process of disaggregation and the sticky nature of polyphenols, fundamental ingredients of such particles.

**Fig. 7:**
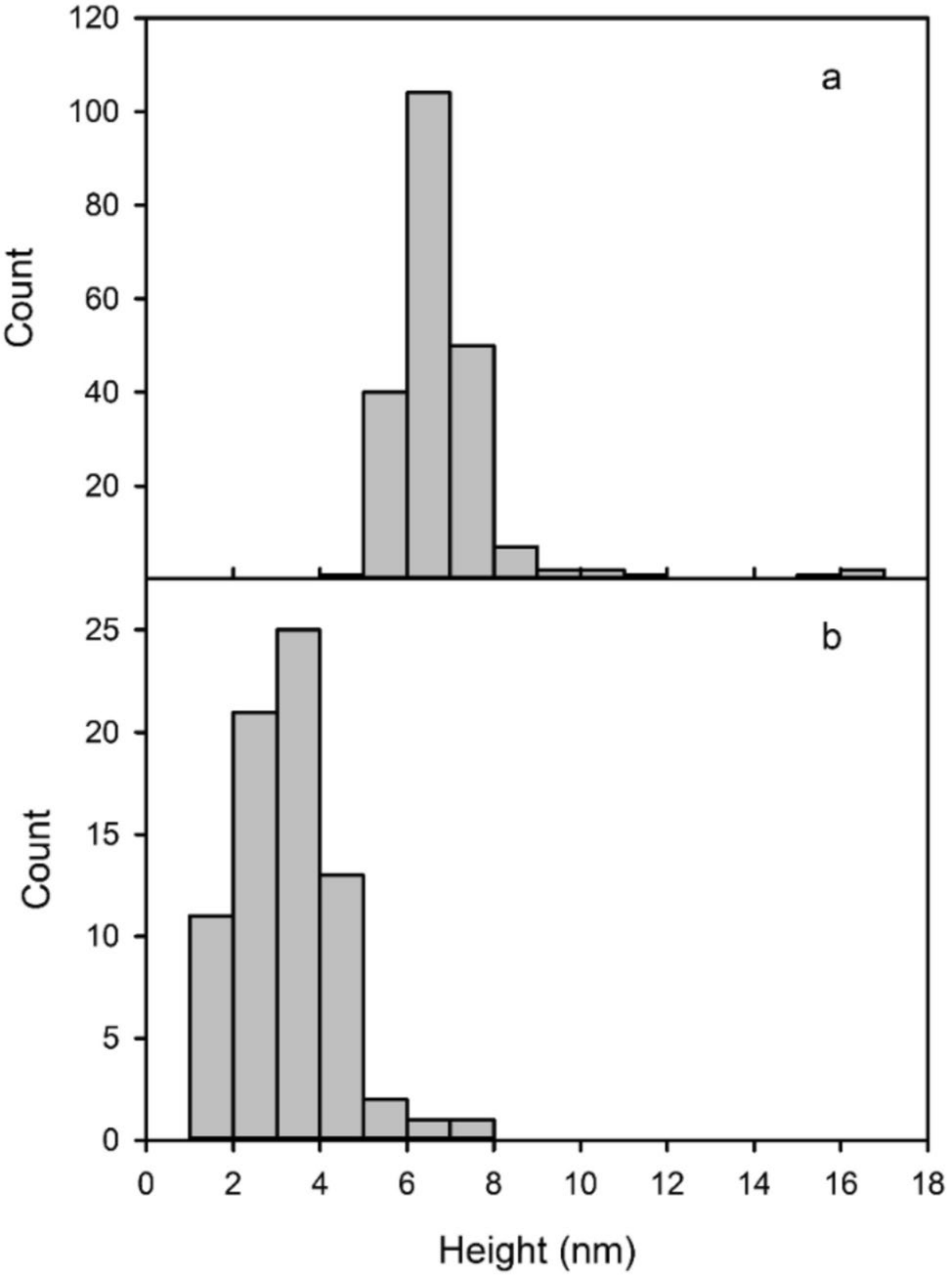
Histogram of particle heights as determined from AFM images for the undiluted and the diluted sample

AFM images were analyzed with a home-made program to obtain the height distribution for the undiluted and diluted extract. Figure 7 shows the resulting distributions. In agreement with light scattering data and coherent with the interpretation of the FC data, the distributions report a decrease of the particles dimension under dilution. In Fig. S2 the images analysis, as reported in Material and Methods section, is shown.

### A simple theoretical model for aggregates disassembly

The experimental data discussed up to this point showed a tendency toward disaggregation of aggregates naturally present in the MO investigated extracts. As a matter of fact, the fragmentation of aggregates, resulting from dilution, increases the solvent-exposed surface area. This could account for the unexpected FC results, specifically the nonlinear trend in polyphenol activity as the extract is diluted. In order to verify if the nonlinearity of FC results can be explained by the fragmentation using simple physico-chemical arguments, the aggregate sizes at different dilutions were modeled by a homogeneous distribution of size, assuming aggregates of spherical shape. We also assume that only the solvent-exposed molecules of such aggregates, simply their area, contribute to the assay reaction. The distribution has been described by a delta-distribution and an associated aggregation number. Thus, the aggregate size distribution can be defined as

1 $$f\left( s \right) = \left\{ {\frac{{N_{{{\mathrm{tot}}}} }}{{s_{0} }}\;\;{\mathrm{if}}\;\;s = s_{0} } \right\}\left\{ {0\quad if\quad s \ne s_{0} } \right\},$$

where *N*_tot_ is the total number of molecules in the sample, *s* is the aggregation number, and ${s}_{0}$ is the average aggregation number.

By approximating the density of the aggregate with average aggregation number ${s}_{0}$ as uniform, then the aggregate occupies a spherical volume with radius proportional to ${{s}_{0 }}^{1/3}$. Then the solvent-exposed surface of an aggregate composed of ${s}_{0}$ molecules scales as the radius squared, i.e., as ${{s}_{0 }}^{2/3}$. If the GAE value under specific sample conditions, i.e., specific dilution, depends essentially on the average exposed area of the aggregates distribution in the sample and on the sample concentration, according to the adopted model, it can be expressed as

2 $${\mathrm{GAE}}\left( {d, N_{{{\mathrm{tot}}}} } \right) = k \mathop \sum \nolimits_{1}^{\infty } s^{{{\raise0.7ex\hbox{$2$} \!\mathord{\left/ {\vphantom {2 3}}\right.\kern-0pt} \!\lower0.7ex\hbox{$3$}}}} f\left( s \right) = k \left[ {s_{0 } \left( d \right)} \right]^{{{\raise0.7ex\hbox{$2$} \!\mathord{\left/ {\vphantom {2 3}}\right.\kern-0pt} \!\lower0.7ex\hbox{$3$}}}} N_{{\text{tot }}} \left( d \right)/s_{0 } \left( d \right) = k N_{{{\mathrm{tot}}}} \left( d \right)/\left[ {s_{0 } \left( d \right)} \right]^{{{\raise0.7ex\hbox{$2$} \!\mathord{\left/ {\vphantom {2 3}}\right.\kern-0pt} \!\lower0.7ex\hbox{$3$}}}} ,$$

where *k* is an experimental constant and *d* is the dilution factor.

The expression contains both the dependence on the size distribution and the concentration of the particles in the sample, stated in $N_{{{\mathrm{tot}}}}$.

The results in Figs. 2 and 3 are normalized for dilution factor *d*, in order to keep constant the samples concentration, $N_{{{\mathrm{tot}}}} *d = N_{0}$.

3 $${\mathrm{GAE}}_{{{\mathrm{norm}}}} = \frac{{kN_{0} }}{{\left[ {s_{0 } \left( d \right)} \right]^{{{\raise0.7ex\hbox{$2$} \!\mathord{\left/ {\vphantom {2 3}}\right.\kern-0pt} \!\lower0.7ex\hbox{$3$}}}} }}.$$

**Fig. 8:**
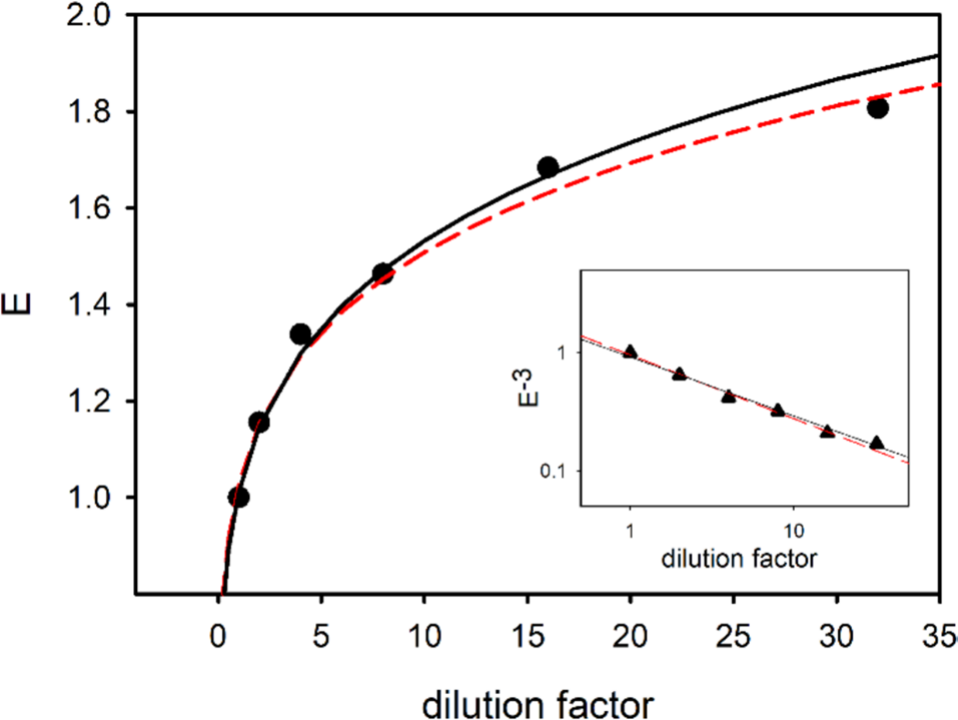
Relative growth of the normalized gallic acid equivalent E(*d*). The lines represent the functions obtained considering a power law dependency of the average aggregate size vs. dilution, with $\beta =-0.54$(black line) and $\beta =-0.5$ (red line). Data and relative analysis of the average aggregate size vs. dilution is reported in the inset

Figure 8 reports the plot of $E\left(d\right)$, that is the relative growth of the normalized gallic acid equivalent, as expressed by the following expression:

4 $$E\left( d \right) \equiv \frac{{{\mathrm{GAE}}_{{{\mathrm{norm}}}} \left( {s_{0} \left( d \right)} \right)}}{{{\mathrm{GAE}}\left( {s_{0} \left( 1 \right)} \right)}} = \left( {\frac{{s_{0} \left( 1 \right)}}{{s_{0} \left( d \right)}}} \right)^{{{\raise0.7ex\hbox{$1$} \!\mathord{\left/ {\vphantom {1 3}}\right.\kern-0pt} \!\lower0.7ex\hbox{$3$}}}} .$$

By plotting ${s}_{0}\left(d\right)/{s}_{0}\left(1\right)={E(d)}^{-3}$ against *d* in log–log scale, data follow a linear trend and can well be interpolated by a power law function, i.e., $\frac{{s}_{0}\left(d\right)}{{s}_{0}(1)}\propto {d}^{\beta }$. By data fitting the value $\beta$ was obtained $\beta =-0.54\pm 0.03$.

The representative aggregation number decreases then with increasing dilution with about the square root of the dilution factor, and therefore, the characteristic size D of the aggregate follows about the relation:

5 $$\frac{{{\mathrm{D}}\left( d \right)}}{{{\mathrm{D}}\left( 1 \right)}} = \left( {\frac{{s_{0} \left( d \right)}}{{s_{0} \left( 1 \right)}}} \right)^{{{\raise0.7ex\hbox{$1$} \!\mathord{\left/ {\vphantom {1 3}}\right.\kern-0pt} \!\lower0.7ex\hbox{$3$}}}} \sim d^{{{\raise0.7ex\hbox{${ - 1}$} \!\mathord{\left/ {\vphantom {{ - 1} 6}}\right.\kern-0pt} \!\lower0.7ex\hbox{$6$}}}} .$$

In terms of the aggregate size, this means that at 32-fold dilution, the uniform size distribution model predicts aggregates with a diameter approximately 0.56 times that of the undiluted sample. Similarly, for the 300-fold dilution explored in the AFM experiment, the predicted average diameter is about 0.4 times that of the undiluted sample. As a matter of fact, an evident asymptotic trend can be noticed by dilution increase in the FC data, suggesting that such effect becomes lower and lower. Although quite simplified, this empirical model well reflects the size distribution change obtained from the in-liquid AFM images and agrees with the initial hypothesis that polyphenols organization can influence the determination of GAE values.

## Discussion

**Fig. 9:**
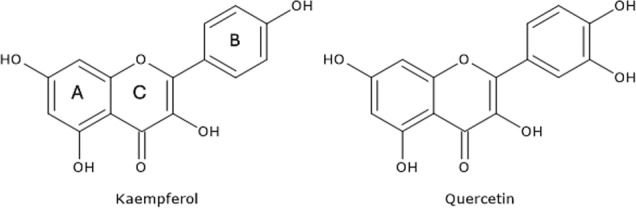
Chemical structure of the two polyphenols, kaempferol and quercetin

In this study we focus on the investigation of Moringa oleifera aqueous extracts structural organization at different dilution values. In our previous work chemical composition, antioxidative/antiradical properties of extracts obtained with different extraction protocols were investigated and the protective effects against typical events on AD were supported by biophysical study and cell test. ORAC and FC assays showed the high antioxidative and antiradical properties of the extracts. The analytical characterization by HPLC–MS identified the rich polyphenolic fraction with quercetin and kaempferol derivatives as main constituents (Carrotta et al. 2025). The effect of MO extracts as inhibitors of A-beta aggregation pointed out the tendency of polyphenols to self-assembling. Indeed, numerous scientific studies have emphasized the remarkable ability of polyphenols to assemble into nanostructures with different sizes, shapes, and chemical characteristics (Guo et al. 2016, 2021; Xiang et al. 2017; Zhou et al. 2020; Yin et al. 2024). In fact, due to their functional phenolic hydroxyl groups, polyphenols can engage in covalent and noncovalent interactions to form supramolecular self-assembled structures (Yin et al. 2024). The main aggregating forces driving self-association are hydrogen bonding, *π*–*π* stacking, hydrophobic interactions, Van der Waals forces, and ionic interactions. In the present work, prompted by the previous one, the morphological characterization, the stability, and the antioxidant availability of particles in the extracts were investigated with FC assay, in-liquid AFM, DLS, and MALS measurements. Light scattering and AFM experiments (Figs. 5, 6, 7) show that polyphenols are organized in aggregates, characterized by a metastable nature. Under dilution in fact, they fall apart, never reaching, as far as we could observe, a completely dissolved state. Separation of the extract in fractions with a 3KDa cut-off allowed to obtain information about the homogeneity of the species in the sample, though having different size. In fact, both fractions present an UV–vis absorption spectrum (Fig. 4) with similar bands, coherent with the main components found by HPLC–MS data (Carrotta et al. 2025). To be more precise, both quercetin and kaempferol due to their structure (they differ only for the R4 hydroxide group) show two bands in the near UV region with maxima at about 270 nm and 370 nm. The long wavelength absorption band is generally assigned to the B- and C- rings as defined in Fig. 9, due to a HOMO–LUMO electronic transition between delocalized molecular orbitals. The higher energy absorption band is instead attributed to the A ring, being well described by a HOMO–LUMO + 1 (with higher energy than LUMO) (Anouar et al. 2012). Spectra analysis evidences that polyphenols appear homogeneously distributed among the differently sized species. As a matter of fact, the fragmentation of aggregates, associated with the dilution, leads to an increased solvent-exposed surface. This would explain the unexpected FC results, i.e., the nonlinear trend of polyphenol antioxidant activity under dilution of the extract. FC method is the most used reference method to standardize the antioxidant power of a solution with unknown composition (Pérez et al. 2023). Despite its widespread use, the FC assay has certain limitations. As an example, the antioxidant activity of polyphenols compounds is influenced by their overall molecular structure (Bors Wolf and Heller 1990; Platzer et al. 2021). Moreover, the test is not entirely specific to polyphenols, as other reducing agents, such as ascorbic acid and certain amino acids, can also contribute to the colorimetric response (Georgé et al. 2005; Bastola et al. 2017). Nevertheless, some authors have studied the use of different methods to clean up the interference substances and alternative FC reacting conditions to limit TPC overestimation (Georgé et al. 2005; Carmona-Hernandez et al. 2021). However, due to its simplicity, sensitivity, cost-effectively, and reproducibility, it remains one of the most commonly used methods for evaluating the antioxidant capacity of food and plant extracts. In our study, for the first time, we observed an unusual nonlinear behavior of FC matching with the aggregation state of polyphenols as determined by AFM and LS investigation. In fact, FC assay showed a clear nonlinear trend, demonstrated in the increase of concentration-normalized GAE value as dilution increase (Fig. 2).

The FC assay of the 3 KDa filtrate sample confirms that also smaller aggregates together with larger ones (Fig. 3) give the peculiar nonlinearity such as the total extract sample (Fig. 2). Put together these results suggest a self-similar structure organization in the polyphenol assemblies in MO extract. Thus, the nonlinear behavior could be explained by considering that assembled polyphenols have a lower capacity to react as an antioxidant. Thus, the fragmentation of aggregates relied to sample dilution causes an increase of polyphenols disposability to counteract the reductive reaction of FC reagent. This hypothesis was confirmed by a theoretical simple model, where the aggregate sizes at different dilutions were modeled by a homogeneous distribution of size, assuming aggregates of spherical shape. Although somewhat simplified, this empirical model accurately reflects the change in size distribution observed from the in-liquid AFM images and supports the initial hypothesis that the organization of polyphenols can influence the GAE determination.

## Conclusions

In the recent decades the discovery of the potential health benefits of natural molecules, inherently present in food, has driven research on natural extracts and their applications in the nutraceutical and pharmaceutical industries, as well as in material science. Moreover, increasing attention has been given to the recovery of valuable compounds from the food industry by-products. In this study, the morphological and structural characterization of the aqueous extract from Moringa oleifera leaves revealed the presence of aggregates that spontaneously form during extraction at room temperature due to the chemical structure of polyphenols. Their self-assembling ability is particularly intriguing, as these spontaneously formed aggregates can serve as a reservoir of antioxidant molecules, readily available for use. From a biotechnological perspective, these nanoparticles could be stabilized and utilized both for their antioxidant properties and as protective carriers for other encapsulated molecules.

## Supplementary Information

Below is the link to the electronic supplementary material.

Supplementary file1 (DOCX 239 KB)

## Data Availability

The data that support the findings of this study are available on request from the corresponding authors.
