# Supplementary material for: Biophysical characterization of polyphenol aggregates in Moringa oleifera leaves water extract: stability and surface exposure effect on antioxidant activity under dilution
Source: Eur Biophys J. 2025 Sep 3;55(2):283–92. doi: 10.1007/s00249-025-01786-4 (PMC13109185; doi:10.1007/s00249-025-01786-4)
Supplement: Supplementary file 1 — Supplementary file1 (DOCX 239 KB) [file 249_2025_1786_MOESM1_ESM.docx]

*Supplementary Information:*

**Biophysical characterization of polyphenol aggregates in *Moringa oleifera* leaves water extract: stability and surface exposure effect on antioxidant activity under dilution**

**Rita Carrotta*, Fabio Librizzi, Vincenzo Martorana, Samuele Raccosta, Maria Rosalia Mangione***

*Institute of Biophysics, National Research Council, Palermo, Italy*

**Corresponding authors: Rita Carrotta (rita.carrotta@cnr.it), Maria Rosalia Mangione (mariarosalia.mangione@cnr.it)*

Fig.S1 shows the representative AFM scan for 300x diluted sample with the same color height scale as Fig. 6a


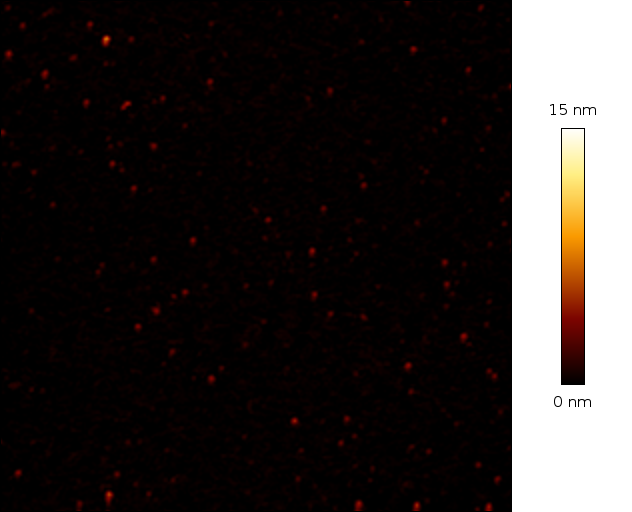


**Fig. S1** 2μm x 2μm image for 300x diluted sample scan.


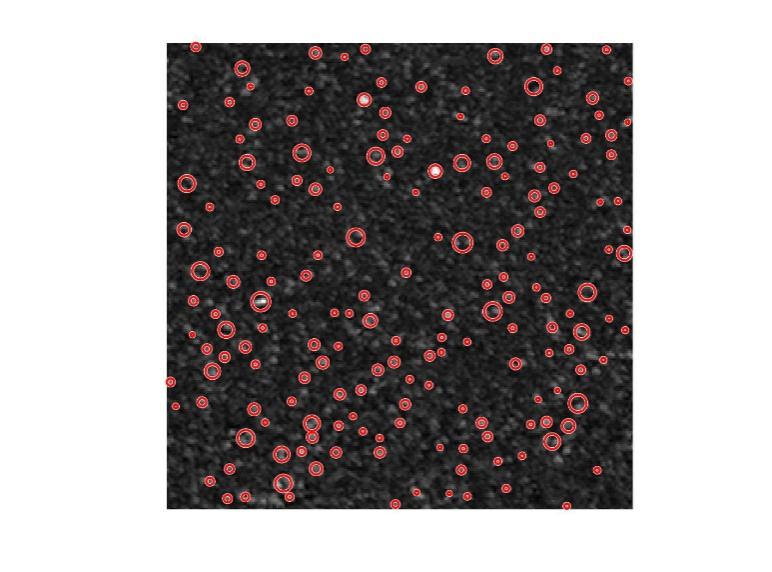

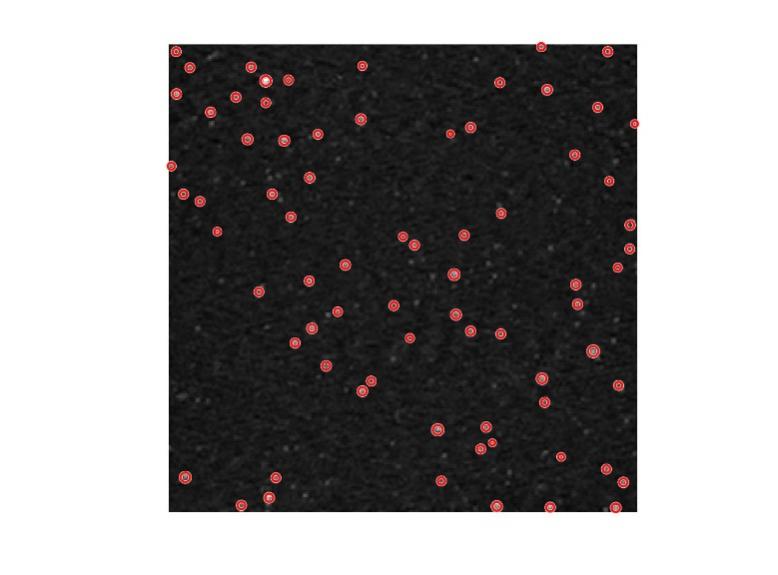
Fig. S2 reports the detection of circular objects in gray scale AFM images

**Fig. S2** Detected circular objects (red circles) both for the undiluted (left) and the diluted sample (right)
